# Neuro-glial interactions at the nodes of Ranvier: implication in health and diseases

**DOI:** 10.3389/fncel.2013.00196

**Published:** 2013-10-29

**Authors:** Catherine Faivre-Sarrailh, Jérôme J. Devaux

**Affiliations:** Aix-Marseille Université, CNRS, CRN2M-UMR7286Marseille, France

**Keywords:** node of Ranvier, ion channel, axon-glial interactions, neurological disease, extracellular matrix

## Abstract

Specific cell adhesion molecules (CAMs) are dedicated to the formation of axo-glial contacts at the nodes of Ranvier of myelinated axons. They play a central role in the organization and maintenance of the axonal domains: the node, paranode, and juxtaparanode. In particular, CAMs are essential for the accumulation of voltage-gated sodium channels at the nodal gap that ensures the rapid and saltatory propagation of the action potentials (APs). The mechanisms regulating node formation are distinct in the central and peripheral nervous systems, and recent studies have highlighted the relative contribution of paranodal junctions and nodal extracellular matrix. In addition, CAMs at the juxtaparanodal domains mediate the clustering of voltage-gated potassium channels which regulate the axonal excitability. In several human pathologies, the axo-glial contacts are altered leading to disruption of the nodes of Ranvier or mis-localization of the ion channels along the axons. Node alterations and the failure of APs to propagate correctly from nodes to nodes along the axons both contribute to the disabilities in demyelinating diseases. This article reviews the mechanisms regulating the association of the axo-glial complexes and the role of CAMs in inherited and acquired neurological diseases.

## INTRODUCTION

In vertebrate, most axons are insulated by myelin sheaths and the action potentials (APs) are regenerated at the nodes of Ranvier which enable the rapid saltatory propagation of the nerve impulses. The myelin is formed by glial cells: Schwann cells in peripheral nervous system (PNS) and oligodendrocytes in central nervous system (CNS). The mechanisms underlying myelin formation are complex and involve interactions between neurons and glial cells. Myelination begins with the contact and recognition of the axon by the glial processes. The glial processes then wrap around the axon, form multiple layers of myelin, and elongate along the axon. Simultaneously, the myelinating glial cells organize the axonal domains: nodes, paranodes, and juxtaparanodes. First, the voltage-gated Na^+^ (Nav) channels are aggregated at hemi-nodes which border the myelinated segments (Vabnick et al., 1996). These hemi-nodes then fuse into a node of Ranvier as the myelin segments grow and approach each over (See **Figure 2**). At both sides of the nodes, the myelin spirals around the axon forming paranodal loops. Electron microscopic observations revealed that the paranodal loops form septate-like junctions with the axon (Einheber et al., 1997). These junctions may preclude current leakage across the paranodes and favor rapid propagation. Recent evidences indicate that the association of Contactin-1/Caspr-1/Neurofascin-155 (NF155) is required for the formation of the septate-like junctions. In addition, these junctions favor the sequestration of the voltage-gated potassium channels (VGKCs; Kv), Kv1.1/Kv1.2/Kv1.6, in the juxtaparanodal regions (Vabnick et al., 1999). The localization of the Nav and Kv channels is strongly dependent on cell adhesion molecules (CAMs) at nodes, paranodes, and juxtaparanodes. Alterations of the axo-glial interaction contribute to the etiology of numerous neurological diseases. This article reviews recent findings documenting the implication of CAMs in axon specialization and in neurological diseases.

## MOLECULAR ORGANIZATION OF THE AXONAL DOMAINS OF MYELINATED FIBERS

### NEUROFASCIN-186, NrCAM, AND GLIOMEDIN: STRUCTURE AND FUNCTION AT PNS NODES

During development, the clustering of Nav is strongly dependent on the axo-glial contact at PNS nodes of Ranvier (Melendez-Vasquez et al., 2001), but also on two scaffolding proteins, ankyrin-G and βIV-spectrin, which links the nodal proteins to the actin cytoskeleton (Jenkins and Bennett, 2002; Komada and Soriano, 2002; Yang et al., 2004; Devaux, 2010). In the PNS, the myelinating Schwann cells form the nodal microvilli which face the nodes of Ranvier. Several CAMs expressed at nodal axolemma or secreted by Schwann cells at the nodal lumen mediate the axo-glial contact and the clustering of Nav channels (Nav1.2 and Nav1.6) at nodes of Ranvier (Caldwell et al., 2000; Boiko et al., 2001). Neurofascin-186 (NF186) and NrCAM belong to the L1-family of CAMs and are concentrated at the nodes of Ranvier (Davis et al., 1996). NF186 is expressed at the nodal axolemma only. By contrast, NrCAM exists as both an axonal form and a form secreted by the Schwann cell microvilli (Feinberg et al., 2010).

**FIGURE 1:**
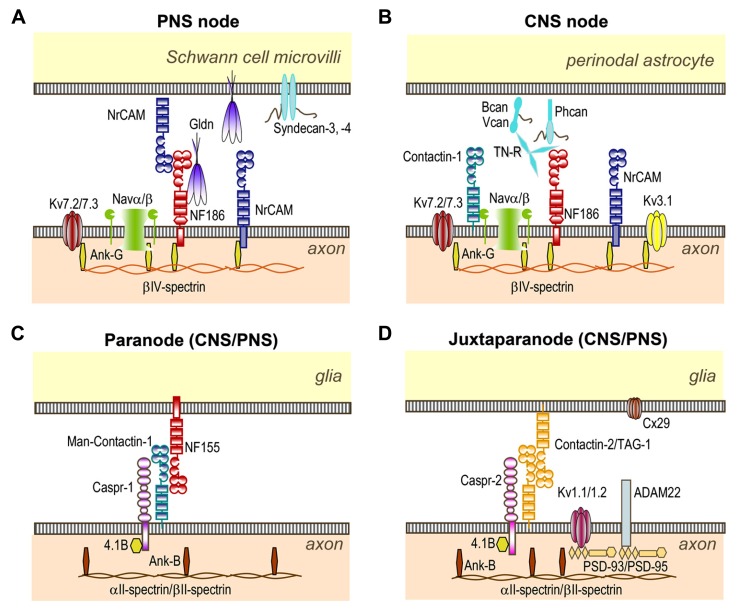
**Organization of CNS and PNS nodes of Ranvier.** **(A)** At PNS nodes, NF186 binds Gliomedin (Gldn) and NrCAM which are secreted by Schwann cells in the nodal gap lumen. The cytoplasmic region of axonal NF186 and NrCAM bind ankyrin-G, which anchors the nodal complex to βIV-spectrin and to the actin cytoskeleton. Ankyrin-G enables the clustering of Nav and Kv7.2/7.3 channels at nodes. **(B)** In the CNS, Tenascin-R (TN-R), Brevican (Bcan), Versican (Vcan), and Phosphacan (Phcan) are enriched in the extracellular matrix surrounding the nodes, and stabilize the nodal complex. These molecules bind NF186, NrCAM, and Contactin-1 which are expressed at CNS nodes. **(C)** The complex Contactin-1/Caspr-1/NF155 forms the septate-like junctions at both PNS and CNS paranodes. This complex is stabilized by the cytosolic protein 4.1B which co-localizes with ankyrin-B, αII- and βII-spectrin at both paranodes and juxtaparanodes. **(D)** The complex Contactin-2/Caspr-2 enables the sequestration of Kv1.1/Kv1.2/Kv1.6 channels at juxtaparanodes, but also of PSD-93 and PSD-95. ADAM22 and Connexin-29 (Cx29) are also enriched at juxtaparanodes.

**FIGURE 2:**
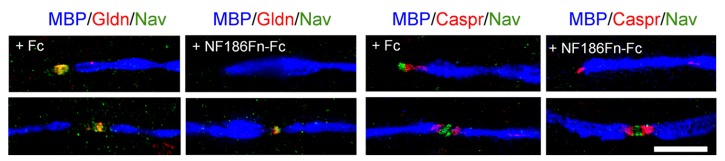
**Soluble FnIII domains of NF186 inhibit the clustering of Gliomedin and Nav channels at hemi-nodes.** These are PNS myelinating co-cultures of DRG neurons with Schwann cells that have been triple-stained for MBP (blue), Caspr or Gliomedin (red), and Nav channels (green). Myelination was induced with ascorbic acid after 7 days *in vitro*. Co-cultures were treated with control Fc or with the FnIII domains of NF186 fused with Fc (NF186Fn-Fc) from day 7 to day 24. Gliomedin (Gldn) and Nav channels are clustered at hemi-nodes and flanked the paranodes and myelin borders in myelinating co-cultures. Incubation with NF186Fn-Fc abrogated the clustering of Gliomedin and Nav channels at hemi-nodes, but not at mature nodes of Ranvier. This indicated that the interaction between NF186 and Gliomedin is crucial for the formation of hemi-node clusters. Scale bar: 10 μm. Adapted from Labasque et al. (2011).

 Both NF186 and NrCAM bind Gliomedin, an extracellular matrix component secreted by the Schwann cell microvilli (**Figure 1A**). Gliomedin contains a coiled-coil, two collagen-like, and one olfactomedin domain (Eshed et al., 2005). Gliomedin exists as both transmembrane and secreted forms (Eshed et al., 2007; Maertens et al., 2007). However, solely the secreted form, generated by proteolytic cleavage with furin and BMP-1 enzymes, is detected at the nodes of Ranvier. The release of the C-terminal olfactomedin domain favors its oligomerization, its incorporation in the extracellular matrix, and its interaction with NF186. The interactions between Gliomedin, NF186, and NrCAM are critical for the initial clustering of the Nav channels at hemi-nodes. In the developing sciatic nerve or in myelinating co-cultures of dorsal root ganglion (DRG) with Schwann cells, the clustering of nodal components (Nav channels, ankyrin-G, NF186, NrCAM, and Gliomedin) is first detected at hemi-nodes at the edge of each myelinated segment (See **Figure 2**). Deficiency in Gliomedin, NF186, or NrCAM prevents the initial clustering of the Nav channels at hemi-nodes both *in vivo* and *in vitro *(Feinberg et al., 2010). Nonetheless, Nav channel aggregation is not prevented at mature nodes in Gliomedin- or NrCAM-deficient animals. As detailed below, mature nodes are flanked by paranodal septate junctions that likely mediate a barrier to the lateral diffusion of the nodal components. Thus, the organization of the PNS nodes depends on axo-glial contacts at nodes and paranodes. The role of NF186 in the organization of mature PNS nodes is, however, controversial. Some studies have shown that NF186 is crucial for the formation of PNS nodes (Dzhashiashvili et al., 2007; Thaxton et al., 2011), but others have shown that deleting NF186 does not alter nodal organization which is maintained by paranodal junctions (Sherman et al., 2005; Zonta et al., 2008; Feinberg et al., 2010).

Recent evidences have underpinned the mechanisms regulating the targeting of nodal components at PNS nodes (Zhang et al., 2012). It appears that nodal CAMs (NF186, NrCAM, and Gliomedin) accumulate to nascent nodes from local sources via diffusion trapping. Nav channels and ankyrin-G, by contrast, are transported to the nodes, and show a slow turnover in mature nodes. The exact mechanisms regulating the selective incorporation of the transported proteins at nodes remained, however, to be elucidated.

The nodal CAMs present several interacting modules which participate in the axo-glial contact. NF186 contains a mucin-related domain, three Fibronectin type III (FnIII) and six Ig domains (**Figure 1**). NrCAM is composed of four FnIII and six Ig domains (**Figure 1**). The Ig domains of NrCAM and NF186 are crucial for their heterophilic interaction (Volkmer et al., 1996). Particularly, NF186 interacts with NrCAM in *trans* via its Ig1–4 domains (Labasque et al., 2011). Deletion of the Ig domains of NF186 abolishes its accumulation at nodes (Dzhashiashvili et al., 2007), indicating that the Ig domains are crucial for the targeting at nodes. In addition, the FnIII domains of both NF186 and NrCAM are implicated in Gliomedin binding (Labasque et al., 2011). Soluble FnIII domains of NF186 has been shown to inhibit the clustering of Nav channels at hemi-nodes in myelinating co-cultures (**Figure 2**). This indicates that the nodal complex assemble via multiple locking modules.

Other extracellular matrix components and their receptors may be necessary for the proper formation or stability of the Schwann cell microvilli, such as laminins and dystroglycan. Specific laminin isoforms (α2, α5, γ5) are expressed in the basal lamina above the nodes of Ranvier (Feltri and Wrabetz, 2005). In addition, members of the dystrophin-dystroglycan complex are present at nodes. Mice deficient in laminin-α2 or dystroglycan show severe alteration of microvilli and Nav channel clusters (Saito et al., 2003; Occhi et al., 2005). Similar alterations are also observed in patients with merosin-deficient congenital muscular dystrophy type 1A which is associated with a mutation in the gene encoding laminin-α2 (Occhi et al., 2005). Because Gliomedin and NrCAM are secreted in the extracellular lumen, it is plausible that the extracellular matrix may stabilize the organization of the nodal components. The proteoglycans syndecan-3 and -4 and Perlecan are also enriched in the perinodal processes of Schwann cells early during development (Goutebroze et al., 2003; Melendez-Vasquez et al., 2005; Bangratz et al., 2012). However, the function of these latter components remains to be determined.

### NF186, NrCAM, AND BREVICAN/VERSICAN COMPLEX: STRUCTURE AND FUNCTION AT CNS NODES

At CNS nodes, the molecular mechanisms implicated in the nodal clustering of Nav channels are different from those involved in the PNS. In the CNS, myelin sheaths are produced by oligodendrocytes, and the nodal gap is contacted by perinodal astrocyte processes. In addition, the extracellular matrix in the nodal gap differs from that in the PNS. The CNS nodes express NF186 and NrCAM, but lack Gliomedin (**Figure 1**). The CNS nodal axolemma also expresses a high molecular weight form of Contactin-1 (Rios et al., 2000), an Ig CAM implicated in the assembly of the septate-like junctions at paranodes (see below). In addition, several secreted proteins are found in the perinodal extracellular matrix surrounding the CNS nodes: Tenascin-R, Brevican, Versican, phosphacan, Bral1, and Neurocan (Weber et al., 1999; Bekku et al., 2009; Dours-Zimmermann et al., 2009; Susuki et al., 2013; **Figure 1**). Brevican and Versican are chondroitin-sulfate proteoglycans that bind hyaluronic acid to form a negatively charged complex with Bral1, the brain-specific hyaluronan-binding link protein. Phosphacan is a chondroitin-sulfate protoeoglycan which is the secreted form of the receptor-like protein tyrosine-phosphatase-β, and which binds Tenascin-R and Contactin-1 with high-affinity (Barnea et al., 1994; Grumet et al., 1994; Peles et al., 1995; Revest et al., 1999). Finally, Tenascin-R is a trimeric glycoprotein consisting of EGF-like and FnIII repeats that may act as a cross-linker between proteoglycan complexes, and which is also able to bind Neurofascin and Contactin-1 (Zisch et al., 1992; Volkmer et al., 1998). These negatively charged matrix components may provide a diffusion barrier around the nodes underlying the accumulation of cations during saltatory conduction (Bekku et al., 2010), but also the stabilization of the nodal complex (Susuki et al., 2013).

In contrast to the PNS, the aggregation of the Nav channels at CNS nodes appears subsequently to the formation of the paranodal junctions (Rasband et al., 1999; Jenkins and Bennett, 2002). Disruption of the paranodal junctions in Caspr-1-deficient mice is associated with important abnormalities at CNS nodes, including Nav channels dispersion and persistent expression of the immature Nav1.2 rather than the mature Nav1.6 subunits (Rios et al., 2003). By contrast, PNS node organization is unaffected in these animals. The axo-glial contact at nodes also participates in CNS node formation. Neurofascin-deficient mice, which lack NF186 at nodes and NF155 at paranodes, show disrupted nodal and paranodal complexes at PNS and CNS. Transgenic expression of NF186 in neurons or NF155 in glial cells can rescue the accumulation of Nav channels at CNS nodes in Neurofascin-deficient mice (Zonta et al., 2008). In contrast to the PNS, the partners of NF186 at CNS node are yet unknown. NF186 can bind directly to Bral1, Brevican, Versican, and Tenascin-R (Volkmer et al., 1998; Hedstrom et al., 2007). However, during development, these perinodal matrix components assemble at nodes after the clustering of NF186 and Nav channels in the optic nerve. Thus, these matrix components may rather be implicated in the maintenance of the nodal structure. In keeping, Nav channels are properly clustered at CNS nodes in Tenascin-R-, Versican-, and Bral-1-deficient mice, despite the loss or dispersion of Tenascin-R and Phosphacan at nodes (Weber et al., 1999; Dours-Zimmermann et al., 2009; Bekku et al., 2010). By contrast, the disruption of the paranodal complex and of the perinodal matrix in Caspr-1/Brevican/Versican triple knock-out mice induces a significant decrease in the number of Nav channel clusters (Susuki et al., 2013). These results lead to the suggestion that the formation of the paranodal diffusion barrier is the primary mechanism enabling the clustering of Nav channels at CNS nodes, whereas nodal axo-glial contact may be a secondary mechanism which allows the maintenance of Nav clusters at nodes or their formation in absence of paranodes.

### CASPR-1, CONTACTIN-1, AND NF155: STRUCTURE AND FUNCTION AT PARANODES

A peculiar type of cell-cell junctions named the septate-like junctions are encountered at paranodes in both the CNS and PNS (Einheber et al., 1997). The septate-like junctions seal the terminal loops of myelinated segments to the axolemma on both sides of the nodal gap. These paranodal junctions are characterized by intermembrane transverse bands and derive from an ancestral type of junctions observed in invertebrates, the septate junctions, that provides paracellular barrier between epithelial cells or between glial cells insulating axon fascicles (Hortsch and Margolis, 2003; Faivre-Sarrailh et al., 2004). In vertebrates, the paranodes act as a fence separating the nodal and juxtaparanodal domains enriched in Nav and Kv channels, respectively and as an electrical barrier that promotes AP propagation. The molecular composition of the paranodal junctions consists of a ternary complex of glycoproteins highly conserved during evolution: Caspr-1, Contactin-1, and NF155. Deficiency in either Contactin-1, or Caspr-1, or Neurofascin in mice induces severe neurological defects, disruption of the septate-like junctions, and a reduction of nerve conduction velocity (Bhat et al., 2001; Boyle et al., 2001; Sherman et al., 2005; Zonta et al., 2008; Pillai et al., 2009).

The axonal Caspr-1 and Contactin-1 form *cis*-heteromers that are targeted to the paranodal junctions during myelination and interact in *trans* with the glial expressed NF155 (Rios et al., 2000; Charles et al., 2002). NF155 is a 155-kDa splice variant obtained from the same gene as NF186, but which is expressed only by the myelinating glial cells (Tait et al., 2000). Caspr-1 belongs to the neurexin family and is composed of a discoidin domain, and several laminin-G and EGF-like modules (Menegoz et al., 1997; Peles et al., 1997; **Figure 1**). Caspr-1 contains a cytoplasmic motif for binding to the scaffolding 4.1B protein and co-localizes with ankyrin-B, αII- and βII-spectrin at paranodes (Ogawa et al., 2006). Contactin-1 and NF155 both contain six Ig domains and four FnIII domains (**Figure 1**), however, Contactin-1 is a glycosyl-phosphatidyl-inositol anchored protein. The assembly and targeting of the Caspr-1/Contactin-1/NF155 complex at paranodes is a tightly controlled process. First, Contactin-1 is required for the transport of the Contactin-1/Caspr-1 complex to the axonal membrane (Faivre-Sarrailh et al., 2000). This complex is addressed to the cell surface with ER-type mannose-rich *N*-glycans that favor its interaction with NF155 (Bonnon et al., 2007). In addition, selective modules are required for the association of NF155 with the Contactin-1/Caspr-1 complex. The Ig domains of Contactin-1 mediate its interaction with NF155 and Caspr-1. Also, the Ig domains 5 and 6 of Neurofascin are implicated in its interaction with Contactin-1. Mutant mice with deletion of these Ig domains show a disruption of the paranodal septate-like junctions (Thaxton et al., 2010).

Worth noting, paranodal proteins are lipid raft-associated proteins and this localization may favor the maintenance of paranodal junctions (Ogawa and Rasband, 2009; Labasque and Faivre-Sarrailh, 2010). Indeed, the deletion of MAL, a raft-associated proteolipid, results in the disorganization of the paranodal septate-like junctions (Schaeren-Wiemers et al., 2004). Also, the maintenance of paranodal junctions appears to be dependent on myelin galactolipids (Popko, 2000; Ishibashi et al., 2002). Mice lacking raft gangliosides, notably GM1 and GD1a, show alterations in Caspr-1/NF155 aggregation at paranodes (Susuki et al., 2007a). In mice lacking Caspr-1 or gangliosides, the partition of NF155 into lipid rafts is strongly attenuated.

### CONTACTIN-2 AND CASPR-2 AT JUXTAPARANODES

The juxtaparanodal regions are adjacent to the paranodes and are recovered by compact myelin. The juxtaparanodes are enriched in Shaker-type Kv1 channels, mainly Kv1.1, Kv1.2, and Kv1.6 subunits, but also Kv1.4 in a subtype of sensory fibers (Rasband et al., 1998; Rasband and Trimmer, 2001). These channels may stabilize conduction by dampening repetitive firing and maintaining the internodal resting potential, particularly during development and in small diameter axons (Rasband et al., 1998; Devaux et al., 2002; Devaux and Gow, 2008). A heteromeric complex of Contactin-2 (also known as TAG-1) and Caspr-2 is implicated in the formation of juxtaparanodes in both CNS and PNS (Poliak et al., 2003; Traka et al., 2003). These molecules are homologs of Contactin-1 and Caspr-1, respectively. Contactin-2 is expressed at the axonal and glial membranes at juxtaparanodes and displays homophilic binding activity which mediates adhesive contact. Contactin-2 exists as a glycosyl-phosphatidyl-inositol anchored form, as well as a released form (Furley et al., 1990). Within the axonal membrane, Contactin-2 forms a *cis*-complex with Caspr-2 via its Ig domains which allows the formation of a ternary complex with the glial-secreted Contactin-2 (Savvaki et al., 2010). Disruption of Caspr-2 or Contactin-2 in knock-out mice prevents the accumulation of Kv1 channels at juxtaparanodes and induces their diffusion along the internodes. Albeit, the mis-localization of Kv1 channels does not affect nerve conduction (Poliak et al., 2003; Traka et al., 2003), it was reported that Contactin-2-deficient animals show behavioral deficits and defects in sensori-motor gating and motor coordination (Savvaki et al., 2008). Strikingly, the transgenic expression of Contactin-2 exclusively in oligodendrocytes is sufficient to rescue juxtaparanode formation and the behavioral deficits in Contactin-2-deficient mice (Savvaki et al., 2010). These data highlight the importance of glial-secreted Contactin-2.

A number of scaffolding proteins (4.1B, ankyrin-B, αII- and βII-spectrin) are expressed at juxtaparanodes with Caspr-2, but also at paranodes (Denisenko-Nehrbass et al., 2003; Ogawa et al., 2006). In 4.1B-null mice, the accumulation of Caspr-2, Contactin-2, and Kv1.1/Kv1.2 at juxtaparanodes is abolished, indicating that 4.1B protein is crucial for the formation of juxtaparanodal domains (Horresh et al., 2010; Buttermore et al., 2011; Cifuentes-Diaz et al., 2011a; Einheber et al., 2013). In addition, the membrane-associated guanylate kinases PSD-93 and PSD-95 are concentrated at juxtaparanodes (Ogawa et al., 2010). However, these proteins are not required for Kv1 and Caspr-2 clustering at juxtaparanodes (Horresh et al., 2010; Ogawa et al., 2010).

The juxtaparanodal complex also comprises disintegrin and metalloproteinase 22 (ADAM22). The deletion of ADAM22 results in the loss of PSD-93 and -95 at juxtaparanodes, but does not affect the localization of Kv1 channels and Caspr-2. The exact function of disintegrin and ADAM22 at juxtaparanodes, thus, remains to be determined.

Of interest, the loss of the paranodal septate-like junctions in Caspr-1 and Contactin-1 deficient mice induces the re-location of the juxtaparanodal proteins near the nodes (Bhat et al., 2001; Boyle et al., 2001). The role of 4.1B in paranode formation or maintenance is uncertain. Nonetheless, the transgenic expression of Caspr-1 lacking the 4.1-binding module in Caspr-null mice restores paranode formation, but does not restore the accumulation of Kv1 channels at juxtaparanodes (Horresh et al., 2010). Altogether, these studies indicate that the organization and maintenance of juxtaparanodes depend on the combination of three distinct processes: assembly of an axo-glial complex at juxtaparanodes, the linkage of this complex to the cytoskeleton, and the sequestration of this complex by the paranodal diffusion barrier.

## IMPLICATIONS OF CAMs IN INHERITED AND ACQUIRED NEUROLOGICAL DISORDERS

### NODE ALTERATIONS IN INHERITED DEMYELINATING DISORDERS

Although nodal/paranodal CAMs are not the priming factors in human inherited demyelinating pathologies, it has came to light during the last decade that demyelination not solely affects the biophysical properties of the myelinated axons but also results in the redistribution or disorganization of the nodal and paranodal components. These latter changes likely participate to the conduction deficits and give important clues about the mechanisms dictating node formation or re-formation during remyelination. Here, we will focus on two human pathologies: the demyelinating forms of Charcot-Marie-Tooth (CMT) disease and Pelizaeus–Merzbacher disease.

Charcot–Marie-Tooth type 1 are inherited demyelinating diseases affecting peripheral nerves which are caused in most patients by mutations in *Pmp22 *(CMT1A), *MPZ *(CMT1B), and *GJB1 *genes (CMT1X; see for review Suter and Scherer, 2003). *Trembler-J* mice are an animal model of CMT1A and show a point mutation in *Pmp22* that is also found in a family with CMT1A (Suter et al., 1992; Valentijn et al., 1992). In these animals, peripheral axons show important segmental demyelination, a reduction in the internodal length, but also a shortening of the paranodal regions (Devaux and Scherer, 2005). These latter alterations are associated with abnormally distributed Kv1.1 and Kv1.2 channels which often flank the nodes or diffuse in demyelinated segments. In demyelinated segments, Nav channels do not diffuse along the axons, but remain clustered at hemi-nodes bordering the Schwann cells (Devaux and Scherer, 2005) and co-localize with Gliomedin (our unpublished observations). These results indicate that despite the paranodal alterations and demyelination, the preservation of the axo-glial contact at nodes is sufficient to enable the clustering of Nav channels in these animals. Interestingly, hemi-nodes and nodes contain two unusual subunits, Nav1.8 and Kv3.1b (Devaux and Scherer, 2005), which are normally absent from PNS nodes. Similar alterations were also found in P0-deficient mice, an animal model of CMT1B. In these animals, most axons exhibit disrupted paranodes and abnormally distributed Kv1.1/Kv1.2 channels (Ulzheimer et al., 2004). In addition, Nav1.8 subunits were found co-expressed with Nav1.6 at nodes and hemi-nodes bordering the Schwann cells in P0-deficient mice. Immunohistological studies of skin biopsies from CMT1A and CMT1B patients have further confirmed that such alterations also take place in human patients. Indeed, segmental demyelination, reduction in the internodal length, and paranodal alterations have been documented in these patients (Li et al., 2005; Bai et al., 2006; Saporta et al., 2009). In particular, reorganization of Kv1.1/Kv1.2 channels was observed in CMT1A patients (Li et al., 2005), whereas, aberrant expression of Nav1.8 subunits at nodes was found in CMT1B (Saporta et al., 2009). Altogether, these findings indicate that demyelination and/or remyelination affects the distribution and composition of ion channels in peripheral axons.

Animal models of Pelizaeus–Merzbacher disease have further revealed some of the mechanisms responsible for the maintenance of Nav channel clusters in the CNS. Pelizaeus–Merzbacher disease is a leukodystrophy associated with mutations in the *PLP* gene. *Myelin-deficient* (*md) *rats and *jimpy* mice are animal models of Pelizaeus–Merzbacher disease, and show severe phenotypes caused by mutations in the *PLP* gene. In both strains, severe dysmyelination occurs during the first post-natal weeks due to spontaneous oligodendrocyte cell death (Knapp, 1986; Grinspan et al., 1998). At P21, few myelinated axons are found in the spinal cord of these animals, and are ensheathed by only a few myelin wraps. Nevertheless, Nav channels and ankyrin-G remain clustered at node-like structures, even in regions devoid of oligodendrocytes (Mathis et al., 2001; Arroyo et al., 2002). By contrast, paranodal regions are severely impacted in the spinal cord of these animals. Caspr-1/Contactin-1/NF155 clusters are not detected, and no septate-like junctions are observed by electron microscopy. Hence, the localization of the Kv1.1/Kv1.2 subunits is strongly altered in *md *rats and *jimpy* mice, and Kv1.1/Kv1.2 subunits abutted the node-like clusters of Nav, Kv7.2/Kv7.3, and Kv3.1b channels (Mathis et al., 2001; Arroyo et al., 2002; Devaux et al., 2003, 2004). These results show that node-like clusters of Nav channels can maintain, at least temporarily, in the absence of myelin sheaths and paranodal junctions in *jimpy* and *md* animals. The mechanisms responsible for the maintenance of these node-like structures are, however, unclear. It is plausible that the presence of astrocyte processes contacting the node or the preservation of the extracellular matrix components (Brevican, Phosphacan, and Versican) maintain these node-like clusters.

## ANTIBODIES AGAINST CASPR-2 AND CONTACTIN-2 IN PERIPHERAL NERVE HYPEREXCITABILITY AND AUTOIMMUNE ENCEPHALITIS

Numerous studies have implicated the molecular complex found at juxtaparanodes, named the VGKC complex, as an autoimmune target in generalized neuromyotonia (Isaac’s syndrome), persistent facial myokymia, Morvan’s syndrome, and in limbic encephalitis. Neuromyotonia and myokymia are peripheral nerve hyperexcitabilities characterized by repetitive muscle contractions (Gutmann and Gutmann, 2004). Neuromyotonia and myokymia are often linked to impaired function of the Kv1 channels. Neuromyotonia is also observed in Morvan’s syndrome in which it is associated to confusion, autonomic disturbance, and delirium or insomnia (Newsom-Davis et al., 2003). By contrast, limbic encephalitis are characterized by amnesia, confusion, seizures, and psychosis (Buckley et al., 2001; Vincent et al., 2004). Originally, it was suspected that antibodies targeting Kv1.1/Kv1.2/Kv1.6 subunits may be the causing agents in these disorders (Shillito et al., 1995; Hart et al., 1997; Buckley et al., 2001; Vincent et al., 2004; Kleopa et al., 2006). However, recent investigations revealed that most patients with anti-VGKC-complex antibodies present antibodies against Leucine-rich glioma inactivated 1 (LGI-1), a secreted protein associated with presynaptic Kv1 channels (Irani et al., 2010; Lai et al., 2010). In addition, many patients present antibodies against the juxtaparanodal CAMs: Caspr-2 and Contactin-2 (Irani et al., 2010; Lancaster et al., 2011). These findings further emphasized that axonal CAMs are implicated in excitability disorders. Worth noting, sera from patients with neuromyotonia, Morvan’s syndrome, or limbic encephalitis recognize cell surface antigens and stain the juxtaparanodes in the PNS (Kleopa et al., 2006; Lancaster et al., 2011). In addition, most of these patients responded to immunotherapy (Irani et al., 2010; Lai et al., 2010; Lancaster et al., 2011), suggesting that the autoantibodies are pathogenics and may induce the down-regulation of the Caspr-2/Contactin-2/Kv1 channel complex. In keeping with this view, sera from patients with neuromyotonia and anti-VGKC-complex antibodies significantly decreased the density of the potassium currents in PC-12, NB-1, or CHO-K1 cells expressing Kv1.1/Kv1.6 cells when the cells were incubated for 3 days with the sera (Sonoda et al., 1996; Nagado et al., 1999). However, these sera did not directly block the potassium currents in these cells.

The fact that antibodies to Caspr-2 or Contactin-2 are associated with peripheral nerve hyperexcitabilities originating in motor axons suggest that these antibodies are susceptible to diffuse across the paranodal barrier and act on the juxtaparanodal Kv1 channels. Recent studies indicate that the paranodal regions is not as tightly sealed as originally thought (Devaux and Gow, 2008; Mierzwa et al., 2010), thus it is plausible that serum IgG in patients with Morvan’s syndrome may slowly diffuse toward the juxtaparanodes. However, the exact pathogenic mechanisms remain to be clarified as well as the epitopes recognized by the antibodies. In some patients, antibodies to Caspr-2 are associated with thymomas (Vincent and Irani, 2010), suggesting a reaction against tumor antigens.

### NODAL ALTERATIONS AND AUTOIMMUNITY AGAINST CAMs IN MULTIPLE SCLEROSIS

Multiple sclerosis (MS) is an immune-mediated disease characterized by CNS demyelination, inflammation, axonal degeneration, and cortical lesions which may lead to numbness, paralysis, blindness, and other deficits. Alterations of the nodes of Ranvier have been documented in MS, and Nav channels appear to diffuse along the demyelinated axons in white matter lesions (Moll et al., 1991; Craner et al., 2004; Coman et al., 2006). In addition, the paranodal length is increased within demyelinating lesions, and NF155 immunoreactivity spreads along the internodes, particularly in damaged or stressed axons (Howell et al., 2006). Worth noting, paranodal alterations precede the dismantling of the node, and result in the incursion of the juxtaparanodal Kv1 channels at nodes and paranodes both in MS and in animal models of MS, the experimental autoimmune encephalomyelitis (EAE; Howell et al., 2006; Zoupi et al., 2013). It is very likely that the disruption of the nodal aggregates of Nav channels participates to the conduction and locomotor deficits in MS patients. Similarly, the alterations of the paranodal axo-glial junctions and the redistribution of the Kv1 channels might contribute to the conduction defects. Several mechanisms may be responsible for these alterations. First, microglia infiltration has been found to correlate with nodal and paranodal alterations in MS patients and in EAE (Howell et al., 2010). Particularly, the inhibition of microglia activation minimized the nodal/paranodal alterations in animal model of MS. This indicates that inflammation can participate in MS etiology by affecting node organization.

Secondly, autoimmune attack against the nodal/paranodal compartments may favor node disruption. Autoantibodies against Neurofascin (NF186 and NF155) have been detected in a few patients with MS (Mathey et al., 2007; Elliott et al., 2012). The immunoabsorption of MS sera over immobilized NF155 abolished the demyelinating and axopathic activities of the serum in one patient (Elliott et al., 2012). Hence, antibodies to NF155 may participate to the nodal/paranodal alterations. However, the prevalence of such antibodies appears to be low in MS patients, as three recent studies indicate that Neurofascin is not the dominant target of antibodies in MS (Devaux et al., 2012; Elliott et al., 2012; Kawamura et al., 2013). Interestingly, the prevalence of antibodies against NF155 is very high (86%) in patients presenting combined central and peripheral demyelination (Kawamura et al., 2013). These patients show a good response to intravenous Ig injection (IVIg) and plasma exchange, suggesting that these antibodies may participate in the demyelination process. The passive transfer of anti-NF155 antibodies in rats does not exert pathogenic effects (Lindner et al., 2013). However, the passive transfer of anti-NF186 antibodies in rats exacerbates the clinical signs of EAE and induces axonal loss (Mathey et al., 2007; Lindner et al., 2013). It is thus likely that antibodies to Neurofascin are pathogenics and participate to the etiology of MS and other demyelinating disorders.

In addition to the humoral response, T-cell response against Contactin-2 has also been reported in MS (Derfuss et al., 2009). The adoptive transfer of Contactin-2-reactive T-cells induces EAE in rats characterized by inflammation of the gray matter. Moreover, Contactin-2-reactive T-cells enhance the demyelinating activity of anti-MOG antibodies by damaging the blood-brain barrier. Taken together, these findings suggest that reactive T-cells may contribute to the pathology of MS. It now appears essential to determine whether other axonal or glial CAMs are the targets of autoimmunity in MS.

### AUTOIMMUNITY TO CAMs IN IMMUNE-MEDIATED DEMYELINATING NEUROPATHIES

A large catalog of neurological disorders affecting peripheral nerves is suspected to be immune-mediated. Among these, autoimmune reaction against the nodes of Ranvier is implicated in Guillain–Barré syndrome (GBS) and chronic inflammatory demyelinating polyradiculoneuropathies (CIDP; Santoro et al., 1990; Griffin et al., 1996; Hafer-Macko et al., 1996a, 1996b; Cifuentes-Diaz et al., 2011b). The causes and pathogenesis of GBS and CIDP remain largely unknown. The presence of inflammatory infiltrates, the deposition of IgG and IgM in nerve biopsies, and the response to IVIg and steroids suggest an autoimmune origin (Dalakas and Engel, 1980; Schmidt et al., 1996; Bouchard et al., 1999; also see for review Hughes and Cornblath, 2005; Mehndiratta and Singh, 2007). In particular, the deposition of complement on the abaxonal surface of the Schwann cells in GBS patients (Hafer-Macko et al., 1996b; Lu et al., 2000; Wanschitz et al., 2003) has suggested that the pathology is humorally mediated.

**FIGURE 3:**
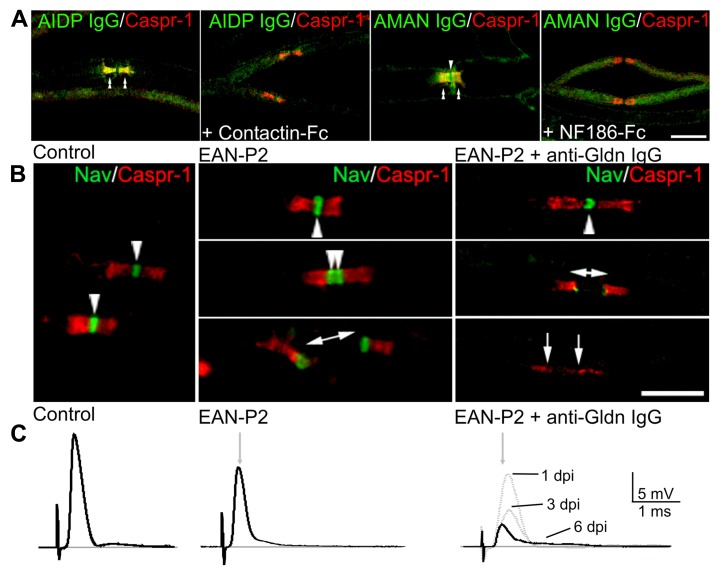
**Antibodies target nodal CAMs in GBS patients and animal models.** **(A)** Mouse sciatic nerve fibers were incubated with sera (green) from AIDP (left panels) or AMAN (right panels) patients which are reactive against Contactin-1 and Neurofascin, respectively. Fibers were stained for Caspr (red) to label the paranodes. Pre-incubation of the sera with soluble Contactin-1-Fc or NF186-Fc abolished the binding of the IgG at nodes (arrowheads) and paranodes (double arrowheads). **(B)** Animal models of GBS were used to evaluate the pathogenic action of anti-Gliomedin antibodies. In animals immunized against P2 peptide (EAN-P2), Nav channels (green) are clustered at nodes (arrowheads) and at hemi-nodes bordering the Schwann cells in demyelinated axons (bar with arrows). The injection of anti-Gliomedin IgG (here 6 days after IgG injection) induces the dispersion of Nav channels in demyelinated segments (between arrows). **(C)** Node disruption is associated with an important conduction slowing and loss in ventral roots of EAN-P2 animals injected with anti-Gliomedin IgG. The amplitude of the nerve potentials progressively decreased 1, 3, and 6 days post-injection (dpi) of anti-Gliomedin IgG. Gray arrows indicate the latency of control nerves. Scale bars: 10 μm. Adapted from Lonigro and Devaux, (2009), Devaux, (2012), and Devaux et al. (2012).

Several recent studies have revealed that autoantibodies in GBS and CIDP patients target CAMs located at the nodes of Ranvier and paranodes (Pruss et al., 2011; Devaux et al., 2012; Ng et al., 2012; Querol et al., 2012; **Figure 3**). In particular, serum IgG in nearly 40 % of GBS and 30% of CIDP patients from a Japanese cohort bind the nodal or paranodal regions of peripheral nerve fibers (Devaux et al., 2012). Also, the serum IgG in nearly 40 % of CIDP patients from a French cohort label the nodal or paranodal regions (our unpublished observations). These results indicate that the node of Ranvier is the target of the immune attack in many GBS and CIDP patients. Gliomedin, Neurofascin, Caspr-1, and Contactin-1 have been identified as the target antigens in some GBS and CIDP patients (Pruss et al., 2011; Devaux et al., 2012; Ng et al., 2012; Querol et al., 2012; **Figure 3**). The proportion of patients with antibodies against these CAMs is relative low and ranges from 1 to 8%. Nevertheless, antibodies to Gliomedin and Contactin-1 are mostly associated with the demyelinating form of GBS, acute inflammatory demyelinating polyneuropathy (AIDP), and with CIDP (Devaux et al., 2012; Querol et al., 2012). Particularly, Querol et al. (2012) have shown that antibodies to Contactin-1 are associated with a specific sub-form of CIDP characterized by an aggressive onset and a poor response to IVIg. In their study, Ng et al. (2012) have examined the prevalence of antibodies against Neurofascin and found that the reactivity against NF155 is more frequent in patients with CIDP. Worth noting, the CIDP patients had IgG4 against NF155. These antibodies may have an antigen-blocking function, as IgG4 does not bind Fc receptors and does not activate the complement pathway (Nirula et al., 2011). Altogether, this suggests that immune attack against nodal or paranodal CAMs could be a common mechanism mediating paranodal demyelination in some sub-forms of demyelinating neuropathies.

Animal models of GBS have further confirmed that autoantibodies to nodal/paranodal CAMs have pathogenic functions. Experimental allergic neuritis (EAN) is induced by immunization of Lewis rats against the P2 peptide (EAN-P2) or purified myelin fraction (EAN-PM) that causes a demyelinating pathology reminiscent of AIDP (Uyemura et al., 1982; Hahn et al., 1988, 1991). Of interest, node disruptions are observed in EAN-PM animals and are associated with antibodies against NF186 and Gliomedin (Lonigro and Devaux, 2009). In these animals, the disappearance of NF186 and Gliomedin at nodes precedes demyelination, and results in the loss of Nav channels in demyelinated segments and in severe conduction defects (Novakovic et al., 1998; Lonigro and Devaux, 2009). By contrast, EAN-P2 animals do not exhibit nodal alterations and antibodies to nodal components, despite the presence of segmental demyelination. This work emphasizes that antibodies to nodal CAMs may participate to conduction defects by dismantling axo-glial attachment at nodes and paranodes. Further, it was found that immunization against Gliomedin, but not NF186, induces a chronic neuropathy with conduction block and nodal dysfunctions (Devaux, 2012). Most importantly, the passive transfer of anti-Gliomedin IgG in EAN-P2 animals induced demyelination, nodal disruption, and an important conduction loss (**Figure 3**; Devaux, 2012). These results showed that primary immune reaction against a nodal CAM can be responsible for the initiation or progression of a demyelinating form of peripheral neuropathy. The passive transfer of antibodies to Neurofascin has also been found to exacerbate the pathology of EAN-P2 (Ng et al., 2012), indicating that these antibodies are pathogenics. In animals injected with anti-Gliomedin IgG, an important deposition of IgG was found at nodes preceding demyelination, but no important deposition of complement (Devaux, 2012). These results suggest that anti-CAMs IgG may induce demyelination by directly blocking the antigen or through the recruitment of macrophages.

The pathogenic mechanisms responsible for the production of anti-CAMs antibodies in GBS and CIDP patients are still elusive. Thus far, no clear correlation has been drawn between infectious agents and the presence of anti-CAMs antibodies. It is worth noting that an outbreak of polyradiculoneuropathy has been reported in a swine abattoir and was caused by aerosolized brain tissue (Meeusen et al., 2012). Nineteen of these patients presented antibodies to the VGKC-complex, and 2 out of 19 recognized Caspr-2. This emphasizes that the mechanisms leading to the production of anti-CAM IgG may be very broad as well as the number of target antigens, and the sub-forms of GBS and CIDP.

### NODAL ALTERATIONS IN IMMUNE-MEDIATED AXONAL NEUROPATHIES

Antibodies against NF186 have also been reported in patients with acute motor axonal neuropathy (AMAN; Devaux et al., 2012). AMAN is the most predominant form of GBS in China and Japan, and is characterized by extensive axonal degeneration. Most patients with AMAN show antibodies against the gangliosides GM1, GD1a, and GalNAc-GD1a (Yuki et al., 1997; Kuwabara et al., 1998; Ho et al., 1999). It is currently suspected that these antibodies bind the nodes of Ranvier and fix complement, then induce node elongation and axonal degeneration (Hafer-Macko et al., 1996a; Paparounas et al., 1999; O’Hanlon et al., 2003). In keeping, rabbits sensitized against GM1 develop an axonal neuropathy similar to AMAN (Susuki et al., 2003). In these animals, the deposition of anti-GM1 antibodies and complement at nodes results in the disruption of the Nav channel clusters and in conduction block (Susuki et al., 2007b). In addition, anti-GD1a antibodies can induce node disruption *in vivo* and *in vitro* (McGonigal et al., 2010; Susuki et al., 2012). These findings indicate that autoimmune attack against the nodes of Ranvier can induce conduction deficits and cause human neuropathies. Thus far, it is unclear whether anti-NF186 antibodies also participate to the etiology of AMAN. The passive transfer of anti-NF186 IgG has been found to exacerbate the axonal loss in EAE (Mathey et al., 2007; Lindner et al., 2013). Because NF186 is located on the axolemma at PNS nodes, we can suspect that antibodies directed against this protein might also induce nodal disruption and axonal degeneration in peripheral nerves. It is thus plausible that in AMAN patients, a broad immune reaction against nodal glycolipids and glycoproteins is responsible for the pathology.

It is worth noting that many axonal neuropathies are associated with node dysfunctions, and are now classified as nodo-paranodopathies (Uncini et al., 2013). For instance, antibodies to GD1b are associated with acute sensory ataxic neuropathy (Pan et al., 2001; Notturno et al., 2008) and result in nodal disruption and axonal degeneration of sensory axons in rabbits (Susuki et al., 2012). Also, alterations of the nodes of Ranvier have been documented in biopsies from patients with chronic idiopathic axonal polyneuropathies (Cifuentes-Diaz et al., 2011b). It would thus be interesting to determine the prevalence of antibodies against nodal/paranodal CAMs in these, but also in other idiopathic neuropathies.

## CONCLUDING REMARKS

Over the last decade, important works have unraveled the nature of the CAMs underlying the axo-glial contacts at nodes, paranodes, and juxtaparanodes. It appears that CAMs participate in the formation and in the stabilization of the axonal sub-domains in a very complex way, and require the cooperation of intracellular anchoring proteins, signaling molecules, and of the extracellular matrix. In the CNS and PNS, the mechanisms regulating the formation of the nodes are different, albeit the composition of the nodal membrane is very similar.

As reviewed here, the node of Ranvier is the epicenter of numerous neurological disorders. This is not surprising owing to the importance of the nodal and paranodal regions in the propagation of nerve impulse. Subtle changes in the biophysical properties or excitability of nerve fibers are likely to lead to broad neurological symptoms such as pain, numbness, confusion, ataxia, or epilepsy. In addition, immune attack against the nodes of Ranvier might be responsible for conduction loss and paralysis in demyelinating disorders and nodo-paranodopathies. Some of the target antigens have been identified, but many still remain to be unraveled. Future works should investigate the pathogenic mechanisms leading to autoimmunity toward nodal antigens.

## Conflict of Interest Statement

The authors declare that the research was conducted in the absence of any commercial or financial relationships that could be construed as a potential conflict of interest.
